# What makes life for process mining analysts difficult? A reflection of challenges

**DOI:** 10.1007/s10270-023-01134-0

**Published:** 2023-11-17

**Authors:** Lisa Zimmermann, Francesca Zerbato, Barbara Weber

**Affiliations:** Institute of Computer Science, University of St. Gallen, 9000 St. Gallen, Switzerland

**Keywords:** Process mining, Challenges, Mitigation strategies, Process analysis, Work practices

## Abstract

Over the past few years, several software companies have emerged that offer process mining tools to assist enterprises in gaining insights into their process executions. However, the effective application of process mining technologies depends on analysts who need to be proficient in managing process mining projects and providing process insights and improvement opportunities. To contribute to a better understanding of the difficulties encountered by analysts and to pave the way for the development of enhanced and tailored support for them, this work reveals the challenges they perceive in practice. In particular, we identify 23 challenges based on interviews with 41 analysts, which we validate using a questionnaire survey. We provide insights into the relevancy of the process mining challenges and present mitigation strategies applied in practice to overcome them. While mitigation strategies exist, our findings imply the need for further research to provide support for analysts along all phases of process mining projects on the individual level, but also the technical, group, and organizational levels.

## Introduction

The massive digitization of enterprises across all industries has led to exponential growth in the amount of data from which these enterprises can gain valuable insights into their performance and identify improvement potential. Therefore, value-adding data analysis represents a decisive competitive advantage when, for example, customer purchasing behavior, impending machine failures, or employee satisfaction are continuously evaluated and the conclusions drawn from such analyses are put into practice. To this end, organizations rely on data analysts capable of obtaining, preparing, and examining data, creating statistical models, and reporting on the obtained results [16, 51]. In doing so, the analysis of internal processes is of particular importance to companies to avoid unnecessary rework or to identify parts of a process that could be automated [44].

Process mining, as a family of techniques offering algorithms for the data-driven reconstruction of (business) processes, validation of their conformance, or enhancement of existing process models based on real process executions [43], is therefore of high interest for many organizations [50]. A testimony of this interest is the rapid development of the process mining market, including an increasing number of tool vendors and an estimated market growth of $1 billion in 2022 [18].

In the past, academic research has approached process mining primarily from a technical perspective [34]. More recently, the increasing use of process mining in organizations has also led to a growing interest in the organizational perspective, which aims to better understand the implementation of process mining in practice and the associated challenges [13, 29]. However, the increased adoption of process mining has also led to a growing demand to attract analysts to work in the field [26]. Still, efforts to better understand their ways of working [20] and insights into how analysts perform a process mining analysis are limited [54]. In particular, since the concrete needs of analysts and individual entry barriers remain largely unknown, there is still little support for individuals to solve challenges that arise during process mining projects.

In this paper, we aim to address this gap and complement existing process mining research at the technical and organizational level by taking an *individual perspective* [50]. Specifically, to investigate challenges perceived by process analysts, assess their relevancy, and elicit mitigation strategies for coping with them, we raise the following research questions (RQs):

1. **What are the challenges perceived by individual process analysts during a process mining project?**
  This research question is subdivided into 2 sub-questions: **RQ.1.1** focuses on the **identification** of the challenges and **RQ.1.2** on the **validation**. In particular, we want to learn whether the challenges identified in RQ.1.1 are understandable and whether analysts experience them in practice.
2. **Do the discovered process mining challenges differ in their relevancy and in the extent to which experienced process analysts are able to solve them in practice?**
3. **What are mitigation strategies applied in practice to overcome these challenges?**

By answering these RQs, we substantially extend our work in [56], which focused only on RQ.1.1. To address all the RQs, we conducted an interview study and a questionnaire survey. In the interview study, we questioned analysts with different role profiles and levels of expertise about the challenges they perceive. As a result of the interviews, we identified 23 challenges (RQ.1.1). Afterward, we conducted a questionnaire survey in which we presented the discovered challenges to a different group of respondents and assessed whether the challenges were understandable and whether respondents had experienced them in practice, and, if so, in which situations. With these insights, we answered RQ.1.2. Based on the feedback from the survey respondents, we also gained insights into the relevancy of the 23 challenges (RQ.2) and into strategies that are applied in practice to mitigate them (RQ.3).

The remainder of this work is structured as follows. In Sect. 2, we provide the relevant background on process mining. Then, in Sect. 3, we present the method followed to answer the RQs by outlining the design and analysis of both the interview study and the questionnaire survey. In Sect. 4, we provide our findings, and in Sect. 5, we compare them to related work. Ultimately, in Sect. 6, we discuss the findings in the light of the process mining research framework [50] considering missing support for analysts and outline the limitations of our work. Section 7 concludes the paper.

## Background

In this section, we provide an introduction to the topic of process mining (Sect. 2.1) and process mining projects (Sect. 2.2). As this work investigates the perception of individuals working in the field, we introduce basic notions and techniques that are relevant to the findings of the paper.

### An introduction to process mining

Process mining is a research discipline that evolved over the last 20 years, quickly gaining traction in the industry. In the *Process Mining Manifesto* [43], researchers and representatives from industry outlined key principles and goals of process mining as a research field, defining three main types of process mining: (i) discovery; (ii) conformance checking; and (iii) enhancement.

Up to date, *Process Discovery* is the most commonly used type of process mining [17]. Its goal is to reconstruct a process model based on log entries of a process execution. Log entries are, for example, created when business processes are executed in an ERP system in which information records, process execution triggers, or document changes leave digital traces in a database. Process mining techniques can then take these digital traces as input and reconstruct a process model. A key requirement for process mining techniques is the proper formatting of the digital traces in the form of an event log [28]. Each event in the log has to contain a case (i.e., trace) identifier, an event name, and a timestamp, indicating when it occurred [43].

There exists a variety of techniques for the discovery of processes that produce process models in different notations [2, 42]. One commonly used graphical notation for discovered process models is the *directly-follows graph* (DFG), which is produced based on all directly-follows relations apparent in the log [42]. While being simple to generate, the DFG has several shortcomings, including its inability to represent concurrent behavior and the inclusion of non-existent process behavior in the representation [41]. Besides DFGs, process mining techniques allow representing discovered process models in other tree-like notations. Examples of such notations are *Petri nets* that can be, for example, generated by the Alpha algorithm [40], process trees as produced by the Inductive Visual Miner [23], or BPMN models [31], as, for example, derived with the Split miner [3]. Next to these graphical notations, process models can also be described based on constraints about the accepted process behavior. Notations such as *DECLARE* and associated rule mining techniques, such as MINERful [6] or Declare Miner [25], allow the formalization of *declarative models* consisting of constraints about the accepted process behavior [10].

After its introduction and maturation, many companies adopted process mining as it got integrated into “Software-as-a-Service” (SaaS) solutions from vendors such as Celonis,^1^ SAP,^2^ or Microsoft^3^ [18]. But also smaller vendors, specialized in certain domains or in specific discovery techniques are disrupting the market. Examples are DCR,^4^ which is focusing on declarative process mining, or Workfellow,^5^ which offers services for less structured, cross-system activities. Next to the commercial tools, there are also open-source tools (such as ProM [45] or RuM [1] ) and software packages (such as BupaR [15] and PM4Py [5]) that can be leveraged to carry out process mining tasks.

Before we present further details about how process mining projects are conducted, we point the reader to the different levels on which process mining can be looked at. Vom Brocke et al. [50] introduced a research framework, differentiating (i) a *Technical Level*, which describes technical aspects such as the development of tools and algorithms; (ii) an *Individual Level*, where the end-user of the solution (in our case the process mining analyst) is taken into account; (iii) a *Group Level* describing the relevancy of teams and interactions within teams to which we are referring as the general group of stakeholders; (iv) an *Organizational Level*, where the governance of organizations and in particular the one of the process mining functions are addressed; and (v) an *Ecosystem Level* describing inter-organizational relations and the impact of process mining on the platform level.

As already pointed out in Sect. 1, we consider our work to primarily contribute to the individual level, as we focus on the perception of process mining analysts. However, we acknowledge that challenges manifesting at this level can also be influenced by other levels and that our findings might lead to important insights on levels (i) and (iii)–(iv) as individuals use process mining tools in an organizational context while working and interacting with stakeholders. We will therefore discuss the relationships between our findings and the technical, group, and organizational levels in Sect. 6.

### Process mining projects

Several methodologies have been proposed to guide the execution of process mining projects. In their work, Emamjome et al. [12] reviewed and summarized four existing methodologies. According to their summary, a process mining project starts with a *Define Research Question* phase, which is characterized as a scoping phase aimed at formulating questions for the project. Afterward, the *Data Collection* phase follows, in which the data (e.g., log entries) required to answer the research question and mine a process model needs to be identified, understood, and extracted from the system. Oftentimes, this data is not directly provided in the required format. Therefore, the *Data Preprocessing* phase is usually carried out to clean and summarize events into one event log table. The *Mining and Analysis* phase follows, intending to produce meaningful process insights. Process analysis tasks are oftentimes exploratory in nature [54] and require a knowledge-intensive analysis of the process models and context information [51]. After obtaining the results, in the *Stakeholder Evaluation* phase, analysts present their results to stakeholders, gather feedback, and, if needed, plan another analysis iteration. The last step of a process mining project can be an *Implementation* phase, in which results are implemented to improve a process. Emamjome et al. [12] remark that this last step is often out of the scope of a process mining project.

In this work, we use the presented phases to classify the challenges perceived by analysts in Sect. 4.1.

## Method

In this section, we present key aspects of the design and conduct of the interview study and questionnaire survey, covering the data collection and the analysis approach followed to address the three research questions.

**Fig. 1:**
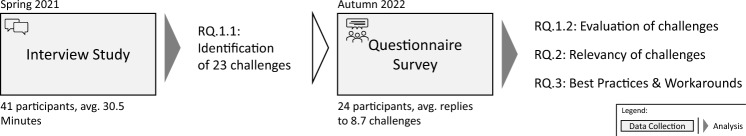
Overview of the conduct and scope of the two studies

Figure 1 shows an overview of our approach. We performed two data collections and the respective analyses following the empirical standards for qualitative surveys and questionnaire surveys [33], respectively. While the focus of the interview study was to answer RQ.1.1, the questionnaire survey was designed to evaluate the findings and, thus, answer RQ.1.2 and gather the additional insights needed to address RQ.2 and RQ.3. More details on the data collections and analyses can be found in the supplementary material published via Zenodo [57].

**Table 1:** Interview study—summary of participant’s roles

	Role description	Count
Academics	Senior Researcher/Professor	12
	Junior Researcher (e.g., PhD Student, Graduate)	8
Practitioners	(Senior-) Consultant & (Senior-) Analysts	11
	Any Business Role (e.g., Process Owner, Operational Excellence Lead)	6
	Product Manager (Tool Vendor)	4

### Interview study

We conducted the interview study as part of a broader data collection having the goal of investigating the concrete work practices of process mining analysts. In that data collection, we first asked analysts to perform a realistic process mining task and analyze a real-life event log, i.e., the road traffic fine management event log [9]. Afterward, we conducted the interviews. As the process mining task is not of particular interest to answer the research questions of this paper, we refrain from providing further details about it. The interested reader may find more information in [55].

*Study Design:* Among others, the interview study was designed to explore the challenges that analysts face when using process mining in practice (RQ.1.1). To gather rich and nuanced insights, we devised a semi-structured interview protocol targeted at a diverse group of process mining experts. The semi-structured design allows adapting the order of questions to the flow of the conversation and to clarify answers when needed. We aimed to prompt experts to reflect on challenges they experienced in the process mining task (i.e., during the analysis of the road traffic fine management event log) and upon general challenges they face in their work practices. In total, we developed five questions to learn about challenges. Of these, the following two are the leading questions. (1) “Is there anything that you found challenging during the task today?”; and (2) “In general, what do you think are the biggest challenges of process mining?”. The full interview protocol can be found in the supplementary material [57].

*Execution and Participants:* We conducted the study via virtual meetings in the spring of 2021. To recruit interviewees, we contacted people in our personal networks and reached out to both, academics and practitioners. We selected participants who (1) had analyzed at least two real-life event logs in the two years prior to the study; (2) were knowledgeable of at least one process mining tool. This ensured that all the participants had hands-on experience with event log analysis. In addition, we asked participants to report on their experience with process mining projects analyzing customer data and excluded those having only experience in process mining research. This allowed us to screen participants based on their practice-oriented experience.

**Fig. 2:**
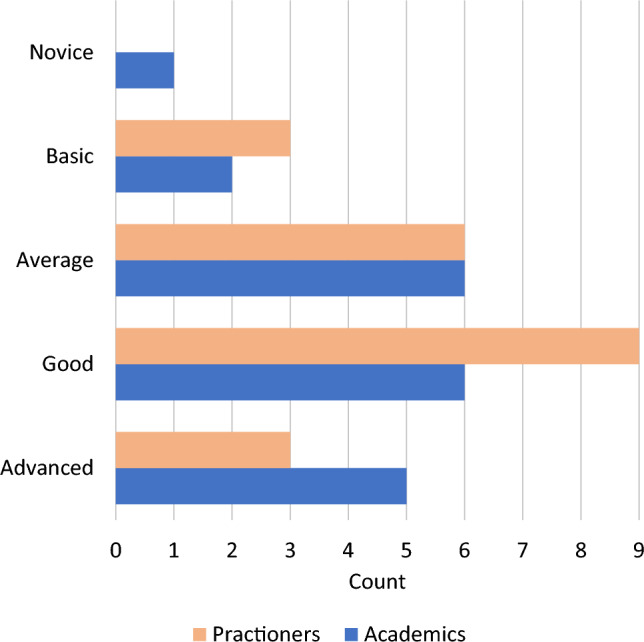
Self-rated expertise Interview study - self-rated expertise

On the day of the meeting, we connected with the participants via video conferencing software and recorded the audio and screen-sharing of sessions. We provided them access to a virtual environment where they first performed the process mining task. Afterward, we conducted the interviews, complementing our interview protocol with questions that prompted participants to describe their work experiences within their current organization.

In total, 41 participants from 27 different organizations took part in the interviews. Table 1 and Fig. 2 show their role description and their level of expertise. On average, the participants had 4.5 years of experience in working with process mining and also indicated experience in data analysis in general. More details about the interview participants can be found in the supplementary material [57].

*Data Analysis:* To prepare the data analysis, we transcribed the full audio recordings. Each session lasted on average 83 min. For this paper, we focused exclusively on the part of the study dedicated to the interviews, which had a mean duration of 30.5 min.

To answer RQ.1.1, we focused on analyzing the interview part about challenges but did not limit ourselves to that, as participants also pointed to challenges while answering other questions. For the analysis, we followed the coding principles of grounded theory [36] and used MAXQDA^6^ as software. Grounded theory provides a systematic method for analyzing unstructured data and allows for detailed insights into our research question [7]. The coding was performed by the first author, while regular alignments on all the identified statements and codes ensured consistency across all authors. We started with “in vivo” and open coding [36] to identify statements about challenges in the interviews. Afterward, axial coding helped us to draw connections between the coded statements and identify categories across participants. Then, we applied selective coding to identify the main concepts until we reached saturation. Ultimately, to keep the focus on relevant aspects, we kept the challenges supported by at least four participants, which resulted in a final set of 23 challenges supported by a total of 371 statements. An example of the coding process can be found in the supplementary material [57].

### Questionnaire survey

To evaluate the findings of the interview study and gain further insights into the challenges, we designed a questionnaire. To address RQ.1.2, RQ.2, and RQ.3, we focus on three main objectives for its design: (i) Evaluate whether the identified challenges are understandable and whether an independent group of respondents can relate to them or has experienced them (cf. RQ.1.2); (ii) obtain information about the perceived relevancy of the challenges and whether they can be solved in practice (cf. RQ.2); and (iii) obtain information about how experts handle situations in which they are confronted with challenges (cf. RQ.3).

**Fig. 3:**
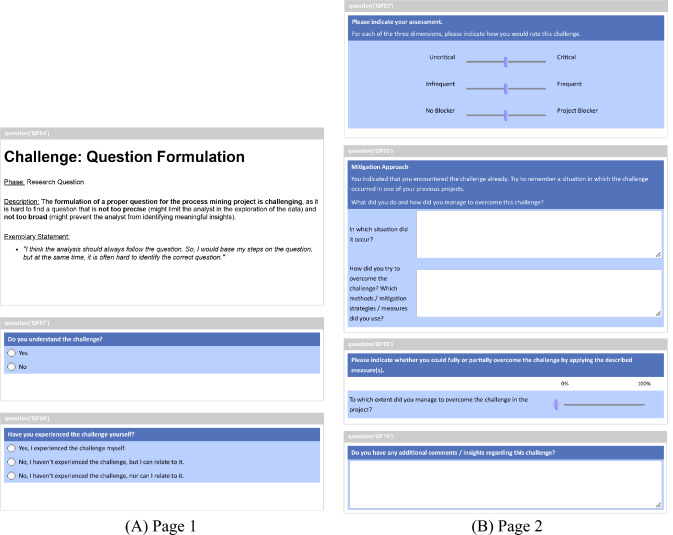
Questionnaire design. Example showing the questions for challenge C1

*Study Design:* To gather information about the three main goals outlined above, we designed a questionnaire including closed and open questions. First, we included demographic questions about the role and previous experience of the respondents. Second, we designed seven detailed questions about the challenges themselves. For their presentation, we provided a description of a challenge similar to the one in [56] but enriched with statements from interviewees. We included the questions separated on two questionnaire pages. An example is shown in Fig. 3.

To validate whether respondents can relate to a challenge, we asked if they (i) understood the challenge; (ii) if they experienced the challenge and, in case they did not experience it; and (iii) whether they can relate to it (Fig. 3A). In case the respondents indicated having experienced the challenge, we additionally asked them to describe the situation in which they encountered it (Fig. 3B). Here, we aimed to retrieve further insights about the challenge itself and the project phases in which it occurs. Further, we included detailed questions to address RQ.2. We asked respondents to rate the relevancy of the challenge on a scale ranging from zero to 100 for three dimensions: (i) criticality; (ii) frequency; and (iii) project blocker. Then, we asked them to indicate (iv) the extent to which they could solve the challenge in their projects along the same scale (Fig. 3B). To address RQ.3, we asked participants to (i) describe the strategies taken to mitigate the challenge and (ii) provide any additional comments (Fig. 3B).

To limit the time and effort for participants, we followed an adaptive questionnaire design and implemented two measures. First, we showed the respondents only the challenges occurring in project phases that they indicated experience in. This prevented respondents from losing time in “skipping” challenges for phases they did not have experience with. In case the respondent indicated experience in more than two project phases, we allowed them to select only the challenges they wanted to give feedback for. Second, we displayed the detailed questions about a challenge (Fig. 3B) only if the respondent indicated experience with it. In this way, we aimed to minimize the effort for respondents and maximize the quality of the replies. For all the challenges, we randomized the order of display to mitigate any bias in the replies based on the order of challenges.

The full questionnaire design can be found in the supplementary material [57].

*Execution and Participants:* The questionnaire survey was carried out in the autumn of 2022. To publish the questionnaire, we used SoSci Survey [24] as the tool guarantees the full anonymity of the respondents and supports randomization and options for the conditional display of questions.

Before publishing the study, we performed a pre-test of the questionnaire with six experienced analysts and academics in our network. Based on their feedback, we implemented the previously described adaptive design and fixed minor presentation aspects. This pre-test also allowed us to obtain a realistic estimate of the time needed to answer the questions, which we could include in the invitation letter we prepared for the respondents.

To distribute the survey, all authors reached out to their professional and personal networks and asked respondents to further distribute the call for participation. We also advertised the survey via social media networks, for example, in the *Process Mining* group on LinkedIn .^7^

To ensure that respondents provided meaningful replies, we required them to (1) have actively participated in at least one process mining project and (2) have at least one year of experience in working with process mining.

**Fig. 4:**
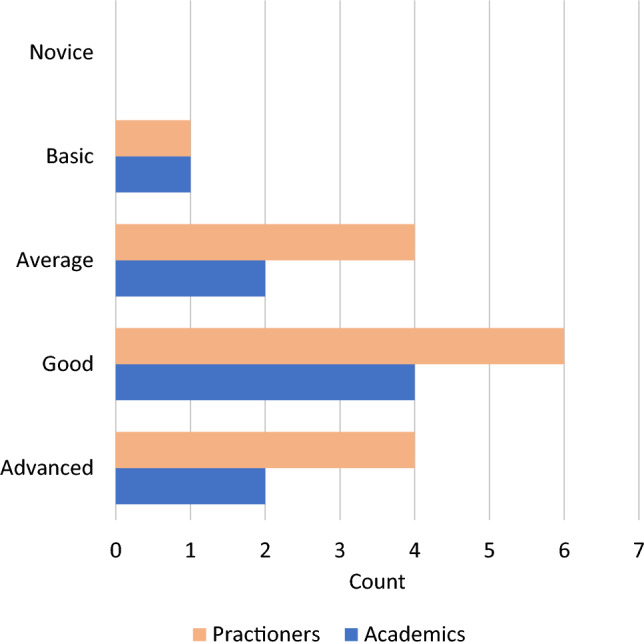
Self-rated expertise Questionnaire survey - self-rated expertise

The data collection lasted from August 2022 to mid-October 2022. During that time, 53 surveys were started, excluding all the website visits without clicks. Of them, 38 answered all the demographic questions. However, 14 dropped out later, as they did not provide feedback on at least one of the challenges (11 respondents) or did not fulfill the inclusion criteria (3 respondents). This resulted in 24 valid replies. We provide an overview of the participants in Table 2 and Fig. 4. Overall, the respondents work in 11 different countries and cover six different job roles, and the majority conducted 3-5 process mining projects prior to the survey. A full list of the questionnaire respondents can be found in the supplementary material [57].

**Table 2:** Questionnaire survey—summary of participant’s roles

	Role description	Count
Academics	Senior Researcher/Professor	2
	Junior Researcher (e.g., PhD Student, Graduate)	4
	N/A	3
Practitioners	(Senior-) Consultant & (Senior-) Analysts	7
	(Senior-) Process Mining Developer	3
	(Senior-) Manager	2
	Transformation Manager/Value Architect	2
	N/A	1

*Data Analysis:* To prepare for the analysis, we created a clean data set containing only replies from the 24 eligible respondents. Note that due to the adaptive questionnaire design, the number of respondents differs for each challenge. On average, each respondent provided feedback for 8.7 challenges.

Below, we describe the analysis approach followed to answer RQ.1.2-RQ.3.

*RQ.1.2:* To validate the challenges derived from the interviews, we analyzed the two questions about whether respondents understood and could relate to the challenge. We considered the relative ratio of respondents that reported understanding and/or having experienced the challenge and the absolute numbers. Since the number of responses per challenge differs, ratios allow for better comparisons across challenges. Additionally, we analyzed the open-text answers reporting on situations in which the challenges occurred to better link situations to project phases. The first author analyzed all the replies and identified relevant patterns based on selective coding [36].

*RQ.2:* To provide insights regarding the perceived relevancy of each challenge, we analyzed the respective numeric ratings per challenge and created an overview of the distribution of replies, considering their means, standard deviations, and correlations. For each respondent who indicated having experienced a challenge, we looked into the following three relevancy dimensions and analyzed whether the challenge was perceived as uncritical (0) vs. critical (100), infrequent (0) vs. frequent (100), and no project blocker (0) vs. project blocker (100).

Additionally, we asked respondents to indicate on a scale from 0% to 100% the extent to which they had been able to solve the challenge in their projects. A rating of, for example, 80% implies that certain aspects of the challenge could be solved but some hurdles remained. For the analysis, we calculated the mean and standard deviations of these ratings.

*RQ.3:* To gather insights into mitigation strategies, we looked into the open-text replies to the corresponding question. For the analysis, we excluded all replies that were too unspecific (e.g., “We added them to do a better analysis.”) or too specific (e.g., “Added a JDBC connection to the satellite systems...”) to be considered as generic mitigation strategies.

Two authors coded the remaining 115 replies independently, using a coding strategy similar to the one described in Sect. 3.1. Several iterations led to saturation between the authors. Based on the coding, five main categories and 13 sub-categories emerged, which allowed us to group the identified mitigation strategies into themes. Since the survey was designed to allow respondents to indicate mitigation strategies for a specific challenge, the strategies were already linked to the challenges when the analysis was conducted.

## Findings

In this section, we present the results of our analysis related to the RQs. We first provide details about the identified challenges and their validity (RQ.1) in Sect. 4.1. Then, in Sect. 4.2, we show the relevancy of the challenges concerning their perceived criticality, their frequency, and their ability to block a process mining project and show to which extent they can be overcome in practice (RQ.2). In Sect. 4.3, we present the strategies that are applied in practice to mitigate the challenges (RQ.3).

### Process mining challenges

Based on the analysis of the interviews, we identified 23 challenges spanning all process mining project phases [12] but the *Implementation* phase (cf. Sect. 2.2). In detail, 19 challenges relate to a specific project phase, while four can occur across phases.

In the following, we describe each challenge and report sample statements from our interviewees. For each challenge, we also present the results of the validation based on the questionnaire survey. In the text, we refer to the participants of the interview study as “participants” or “interviewees” and report the total amount of participants who supported the challenge as #/41, where P = # and A = # indicate“**P**ractitioners,” respectively “**A**cademics.” For the results of the questionnaire survey, we refer to “respondents” and report the number of respondents who provided an assessment of the challenge as R = #/24, using the same style for practitioners and academics. In the summary tables (Tables 3–8), we recall the number of interviewees (Int. No.), the total number of respondents (No.), and the number and ratio of respondents understanding (Und.) and experiencing (Exp.) the challenge.

#### Define research question

Existing methodologies suggest the existence of a planning phase, summarized as *Define Research Question* [12]. Based on the conducted interviews, we identified three distinct challenges for this phase, which were reported by 15/41 (P = 8, A = 7) participants and later evaluated by 12/24 (P = 6, A = 6) respondents. The three challenges are summarized in Table 3.

**Table 3:** Overview of the challenges identified for the *Define Research Question* phase

	ID	Challenge	Description and Quote	Int. No.	Evaluation		
					No.	Und.	Exp.
	C1	Question Formulation	Formulating “good” questions for a process mining project is challenging, as it is hard to find a question that is not too precise (might limit the analyst in the exploration of the data) and not too broad (might prevent the analyst from identifying meaningful insights)	10	9	8 89%	8 89%
			*“I think the analysis should always follow the question.”* [...] *“but at the same time, it is often hard to identify the correct question.”*				
	C2	Access and Use of Process Mining Tools	The access to process mining tools or the choice of which tool to use is challenging, as tools might be unavailable, inconvenient to use, or a governance is missing to structure tool usage in the organization	6	6	5 83%	1 17%
			*“I think the biggest challenge is that most companies do not have the tools implemented.”*				
	C3	Process Mining Suitability	Identifying process mining as a suitable method for the problem or goal at hand is challenging	4	4	4 100%	2 50%
			*“I think it makes a lot of sense to use process mining methods. But at the same time, you don’t need process mining to answer a lot of the questions. You can use process mining as a tool in the toolbox where you have a lot of other tools that you use around.”*				

*C1: Question Formulation.* A fourth of the interviewees (10/41, P = 6, A = 4) mentioned challenges connected to formulating questions or working without clearly formulated questions or goals. It was pointed out that *“it is very often hard to identify the correct question”* for a project and that *“when you don’t have any question you are not focused and your analysis will most likely not be correct.”*

Formulating a question, lacking specifications to identify the *correct* question, or working with a question that is too broad or too narrow was deemed challenging by 88.89% of the survey respondents (R = 9/24, P = 4, A = 5) who indicated having understood and experienced the challenge. They stated that formulating questions *“is a challenge of every process mining or data analytics project”* and that *“there is often no clear-cut question, but rather it is kept open according to the principle ‘see what you find’.”* One respondent summarized the problem: *“Having a clear question helps to target the analysis, but it can also prevent interesting insights from being discovered because certain aspects of the process are excluded from the start.”*

*C2: Access and Use of Process Mining Tools.* This challenge, reported by 6/41 (P = 3, A = 3) interviewees, covers having access to process mining software and using it. Tools are either not implemented in the organization, analysts do not know how to work with a specific tool, or they claim poor usability.

When confronted with it, 83.33% of the respondents (R = 6/24, P = 3, A = 3) understood the challenge, but only 16.67% experienced it. Access to a process mining tool is often a precondition for a project, making it less likely to be experienced by analysts who work in an environment where process mining is already adopted: *“Before starting a process mining project, a fitting tool needs to be identified and bought [...] Also, the governance needs to be established, e.g., roles (engineers, analysts, administrators).”*

*C3: Process Mining Suitability.* Determining whether process mining is suitable for answering a given question is a challenge for 4/41 (P = 3, A = 1) interviewees, as *“for a lot of the questions you don’t need process mining to find an answer.”* They also reported difficulties in identifying process-mining-specific use cases and demonstrating the usefulness of the method to stakeholders: *“It is hard for consultants to convince people that it is something we should have, a new process mining project targeting this and that.”*

The challenge was confirmed by 4/24 (A = 4) survey respondents. Of them, the two respondents who experienced it added that *“stakeholders often don’t care which exact methods are applied; whether these are process mining methods, general data mining methods or even simple calculations in Excel.”*

#### Data collection

The *Data Collection* phase aims to identify, understand, and collect the data for the analysis [12]. Based on the feedback of 23/41 (P = 14, A = 9) participants, we identified four different challenges for this phase, shown in Table  4, which were validated by 18/24 (P = 11, A = 7) experienced analysts.

**Table 4:** Overview of the challenges identified for the *Data Collection* phase

	ID	Challenge	Description and Quote	Int. No.	Evaluation		
					No.	Und.	Exp.
	C4	Data Extraction	The extraction of data is challenging, as problems can occur during the identification and extraction of event data from any source	18	10	10 100%	8 80%
			*“Gathering the data is a technical challenge”* [...] *“You quite often need to extract data from information systems that are not thought to be able to export clean logs.”*				
	C5	Data Availability	Having the required event data available is challenging, as data might not be stored in the source system or is missing because process steps are conducted outside of the system	11	9	9 100%	4 44%
			*“In one of the projects we did not have enough event data because the process was recently changed. And we didn’t have enough cases so we couldn’t continue with the project.”*				
	C6	Data Access	Getting access to the data is challenging, as permissions (legal, regulatory, organizational) or means to access it can be missing	8	9	9 100%	8 89%
			*“Another challenge is to check if we can get the data. Because we already had cases where we couldn’t get the data, it was not accessible, not for me and not for the data scientists.”*				
	C7	Source System and Data Structure Knowledge	Gaining knowledge regarding the source system and the data structure within this system is challenging, as system information and documentation might not be available	6	12	12 100%	10 83%
			*“Most systems require knowledge, and I don’t have that because I’m not an expert on the system and the settings there.”*				

*C4: Data Extraction.* The most significant challenge perceived during the *Data Collection* phase is the extraction of the data itself. 11/41 (P = 6, A = 5) interviewees reported difficulties due to dependencies on external stakeholders or due to the timely nature of this task. Analysts find it difficult to provide clear requirements to their counterparts in the IT departments (*“There is a lot of work to do for explaining to the partners what is really needed from them and what is needed from the data”*) and to receive all the data required for one analysis. Interviewees also remarked that many source systems do not offer out-of-the-box options to extract the data and that methodological support is needed: *“It would be very useful for commonly used information systems to have methodologies and policies in place for the management and collection of data.”*

Out of the 10/24 (P = 6, A = 4) respondents who assessed this challenge in the survey, all understood it and 80% reported having experienced it. Respondents added that *“ETL functionality in process mining tools is lacking. Very often this happens outside of the process mining tools and you are on your own there.”* Moreover, they stressed the technical limitations of source systems: *“The source system allowed only a certain amount of exported rows per day, which was a blocker for the project. In another situation, nobody was allowed to extract data from the system as it was highly fragile.”*

*C5: Data Availability.* Along with data extraction, data availability is another challenge. 9/41 (P = 6, A = 3) participants reported not being able to collect a suitable amount of data for their projects. From the interviews, we identified two main problems related to being *“limited to what is recorded by the system.”* One is the low volume of usable data available: *“We had less or not enough event data to check because the process was changed. And, in a way, we didn’t have that many cases and the data wasn’t enough to say if the process was working or not.”* The other is the absence of data for specific (not recorded) process steps: *“In the end, you have a letter that goes out and, in that case, having a digital footprint of the process is difficult.”*

9/24 respondents (P = 5, A = 4) confirmed understanding the challenge. 44.4% of them reported having experienced the challenge, while 33.3% could relate to it without having experienced it.

*C6: Data Access.* Access to event data may be restricted by regulations, such as the General Data Protection Regulation (GDPR),^8^ which governs the use of personal data, or company-specific compliance policies [13]. Moreover, technical reasons like missing application programming interfaces (APIs) limit direct access to the data. Challenges regarding access to the raw data were mentioned by 6/41 (P = 5, A = 1) of the interviewees. Participants described that *“getting permission to get access to the data is always a struggle”* and that it is challenging to *“disentangle every confidentiality problem that can be raised by the process mining project.”*

9/24 (P = 6, A = 3) respondents understood the challenge, and 88.9% of them indicated having experienced it. According to them, this challenge occurs when *“the company has strong regulatory checks in place”* or when it is *“not possible to connect”* [a system] *“directly with a process mining tool.”*

*C7: Source System and Data Structure Knowledge.* Based on the answers of 4/41 (P = 4) interviewees, we identified that missing knowledge about the source system is problematic, as knowing the structure and the functionality of the source (database) system is a precondition for identifying and understanding the data to collect.

In the survey, both practitioners and academics confirmed this challenge. All of the 12/24 (P = 7, A = 5) respondents providing feedback for it understood the challenge, and 83.3% experienced it. One respondent wrote that *“it occurs every time I need to analyze data from a source system I have never worked with before. There are often no data catalogs or documentation for source systems and their data tables and fields.”* In some cases, *“even the technical staff involved in the management of such systems often does not know exactly where the data is stored.”*

#### Data preprocessing

The third phase of the project involves processing the collected data and transforming it into the required event log format. 24/41 (P = 12, A = 12) interviewees reported challenges connected to the transformation of the data itself but also challenges related to data quality and validation of the (transformed) data. An overview of the three identified challenges can be found in Table 5. These challenges were evaluated by 18/24 (P = 11, A = 7) respondents of the questionnaire survey.

**Table 5:** Overview of the challenges identified for the *Data Preprocessing* phase

	ID	Challenge	Description and Quote	Int. No.	Evaluation		
					No.	Und.	Exp.
	C8	Data Transformation	Transforming the extracted data into an event log is challenging, as it can be problematic to add attributes or activities to the event log and find a good level of abstraction depending on the case key	17	12	12 100%	10 83%
			*“I think the biggest challenge is getting your data in a format that is ready to be analyzed. Typically, data comes from ERP systems like SAP or similar and it’s not straightforward to transform it into an event log or an XES format.”*				
	C9	Data Quality	It is challenging if the quality of the data extracted from the source system does not meet the expectations and analysts need to deal with low quality either in the raw data or the final event log	15	11	11 100%	8 73%
			*“The most problematic challenges are data quality issues. Frequent data quality issues and the mismatch of data recording and the business level reasoning.”*				
	C10	Data Validation	The validation of the data is challenging, as it can be difficult to check the correctness and completeness of the data and its suitability for being analyzed with process mining techniques	5	11	11 100%	7 64%
			*“My experience is that I need a lot of time to check if the data is ok and accurate.”*				

*C8: Data Transformation.* The first challenge identified for the *Data Preprocessing* phase concerns the transformation of the data. It was mentioned by 17/41 (P = 9, A = 8) participants. They reported that it is hard to *“consolidate the data”* and *“define how you want to see the event log.”* The preparation of the event log for the analysis is time-consuming and covers major parts of the project timeline. In particular, analysts seem to struggle with adding attributes to the event log (*“I try to consolidate data sources and add good attributes, but this is the most challenging part.”*), adding activities (*“It feels like a philosophical question of which activities to add.”*), or finding a good level of abstraction for those activities (*“If I select too many process steps and I have hundreds of process steps within the process, then I get complexity problems. This is also a balance you need to find and that people underestimate.”*).

The challenge was confirmed by 12/24 (P = 6, A = 6) respondents of which all indicated understanding the challenge and 83.3% having experienced it in their projects. Experts pointed out that the challenge can occur when dealing with data that were previously extracted from either one or multiple systems, or when it is provided by external project partners.

*C9: Data Quality.* Challenges related to data quality were reported by 15/41 (P = 6, A = 9) participants. While the majority of the 15 participants referred to data quality as a general challenge, more specific problems such as inconsistencies across timestamp formats, missing timestamps, and timestamp synchronization across time zones emerged. Additionally *“noise in the data”* and *“repetitive activities or missing case numbers”* were mentioned.

In the survey, this challenge was confirmed by 11/24 (P = 7, A = 4) respondents. 100% of them understood data quality challenges, and 72.73% of them experienced such challenges during the event log creation.

*C10: Data Validation.* While C9 refers to difficulties in transforming the extracted data with quality issues into a process mining format where those quality issues are resolved, the challenge of data validation refers to the identification of such issues. 5/41 (P = 3, A = 2) participants reported this challenge mainly referring to the event log resulting from the *Data Preprocessing* phase. For example, one interviewee narrated that *“one challenging thing is to identify if you can trust the data. Because if you just get the data and read it in Disco, you don’t know if it is correct because a data scientist makes a query”* [to extract it] *“but then it’s my job to check if I can trust the data, otherwise, I might analyze wrong data. So, I think the biggest challenge is to know that the data is really correct.”*

This challenge was confirmed by almost half of the survey respondents, i.e., 11/24 (P = 9, A = 2). All of them understood the challenge and 63.64% reported having experienced it. While three respondents described the challenge as a general problem that always occurs in their projects, it seems to occur more often in situations where ground truth for the validation is missing: *“It occurred in a project in which we analyzed a very complex process. The reason to use process mining was that there was no transparency before. Hence, there was no report or ground truth regarding the numbers of documents or KPIs to compare the data.”*

#### Mining and analysis

In the *Mining and Analysis* phase, analysts aim to derive answers to a question by applying process mining techniques within commercial or open-source process mining tools [35]. 38/41 (P = 19, A = 19) interviewees reported seven distinct challenges occurring in this phase, which are shown in Table  6. When we presented those challenges to the survey respondents, 18/24 (P = 10, A = 8) provided their assessment.

**Table 6:** Overview of the challenges identified for the *Mining and Analysis* phase

	ID	Challenge	Description and Quote	Int. No.	Evaluation		
					No.	Und.	Exp.
	C11	Tool Knowledge	Having proper knowledge of how to use a tool is challenging, as analysts might not be familiar with the tool, lack knowledge regarding specific functions, or have not used it in a while	18	11	9 82%	6 55%
			*“The tools work all in a very similar way and they basically use the same algorithms. But remembering where those patterns are and how to click in the right sequence is not always easy.”*				
	C12	Event Log and Data Model Understanding	Understanding the event log or the data model is challenging, as they can come in different formats and with different levels of complexity	15	9	8 89%	6 67%
			*“Understanding the data model in the tool is probably the biggest challenge.”*				
	C13	Process Mining Techniques	Process mining tools lack (or insufficiently support) certain process mining functionalities so analysts are not able to solve specific problems with a given tool or are not satisfied with the output of a technique	14	9	8 89%	6 67%
			*“I think solving this analysis problem is challenging, considering there is no methods for that in the tool.”*				
	C14	Access to Additional Information	Access to additional information during the analysis is challenging, as analysts do not have permission to access the process documentation, cannot collaborate with knowledgeable stakeholders (e.g., process owners), or such information does not exist	10	9	8 89%	6 67%
			*“If I don’t have access to additional information, I only get what is obvious from the process.”*				
	C15	Process Visualization	Interpreting processes represented as DFGs is challenging, as they are not able to deal with concurrency and should be cautiously read when filtered	8	11	11 100%	9 82%
			*“There are many challenges” [...]* *“For example, the outcome of discovery algorithms can be imprecise in a way that they generate visualisations with lots of behavior that is not present in the event logs. The notation used for visualizing process models is not always accurate.”*				
	C16	Analysis Experience	Having analysis experience is difficult and performing an analysis can be hard if the analyst does not have a lot of experience. Also, gaining experience and finding project partners with the required skills is challenging	7	6	6 100%	2 33%
			*“The analysts need to be experienced in process mining, and have the correct mindset and understanding for the situation. It is a challenge if this person is not available.”*				
	C17	Analysis Focus	Keeping the focus on the question during the analysis is challenging. It can be problematic to maintain the big picture of the analysis despite the available details	6	8	6 75%	4 50%
			*“The biggest challenge is to not lose yourself in the top-down analysis.”*				

*C11: Tool Knowledge.* The most frequently mentioned challenge in this phase concerns the analyst’s ability to use process mining tools. Although all the interviewees were knowledgeable of one or more tools, 18/41 (P = 8, A = 10) reported *“not feeling comfortable with the tool”* and mentioned difficulties in finding specific functionalities. Using a tool efficiently without being accustomed to using it regularly is challenging. Despite the enhancements routinely implemented by vendors, tool usability seems to be still an issue: *“If you go for ProM, you don’t know what plugins to use. If you go for Celonis, you don’t know how to create a dashboard or you struggle to use PQL. So you cannot do anything, even in Disco, you need to know how to filter.”*

In the survey, 11/24 (P = 8, A = 3) analysts responded to this challenge, out of which 81.82% indicated understanding it and 54.55% having experienced it. While respondents confirmed that the challenge exists, they provided little additional information about the situations in which it occurs.

*C12: Event Log and Data Model Understanding.* Challenges related to understanding the data model, the relations within the data model, specific event log attributes and attribute names, or the event log structure were mentioned by 15/41 (P = 8, A = 7) participants. Difficulties are particularly emphasized when elements one is accustomed to working with are missing from an event log. Existing process mining tools offer different options to load the data. While tools like Disco and ProM allow uploading the event log as one file (e.g., XES or CSV), other tools like Celonis support the creation of data models with a relational structure as the standard. Some of the interviewees highlighted the need for more support in understanding the event log since *”there are a lot of tools for the pre-processing, but there is no support for the understanding. Getting acquainted with the log is difficult.”*

88.89% of the survey respondents (R = 9/24; P = 5, A = 4) indicated understanding the challenge and 66.67% of them having experienced it in their projects. The respondents remarked on the inconsistencies between the tools: *“I’ve worked with Disco, PM4Py, and ProM - each tool converts the data to the event log differently.”* This seems to be particularly challenging in situations where analysts are *“not involved in the data extraction and have no access to domain or database experts.”*

*C13: Process Mining Techniques.* The statements of 14/41 (P = 8, A = 6) of the interviewees refer to missing or insufficiently advanced functionalities in the area of process mining and, in particular, to the lack of implementation of these functionalities in process mining tools. Our analysis shows that although the tools and the functions they support are constantly evolving [17], relevant features are not yet integrated. Participants named challenges revolving around the limited integration of robotic process automation (RPA), limitations of the dotted chart in ProM, the *“inability of any algorithm to split labels based on the context,”* and, predominantly, the lack of support for automated root cause analysis based on event data.

88.89% of the respondents (R =  9/24; P = 3, A = 6) understood the challenge, and 66.67% experienced it. Next to the need for better root cause analysis, missing technical support for *“handling the complexity of the processes,”* *“automated data ingestion,”* *“hierarchical process discovery,”* and *“advanced capabilities for predictive and prescriptive analytics”* were reported.

*C14: Access to Additional Information.* Our participants reported that *“it is often the case that we need some additional information to really get into an event log.”* Such additional information is either documentations or obtained through interactions with domain experts. However, 10/41 (P = 7, A = 3) participants indicated that access to such information can be a challenge. Sometimes, process and/or system documentations do not exist or are not detailed enough to be used in a project: *“Usually, I don’t have much good documentation. I just have the data table and a few indications, which makes it more challenging.”* As part of this challenge, difficulties in accessing domain experts were also noted. For example, participants explained that *“it is hard to have access to those people, to interview them. You can take a look at the data and the process, but the interviews help you to analyze better and guess better what is relevant.”*

During the validation, 88.89% of the respondents (R = 9/24; P = 5, A = 4) confirmed understanding the challenge and 66.67% experienced it. We learned that the challenge might occur *“when working with public data where there is no access to further information or domain experts. Other projects could usually be solved by talking with the experts.”* But also in contexts where the data of an organization is analyzed, access to the stakeholder is not always given: *“We had a request to add non-standard activities to a process but we received no further information from the process owner.”*

*C15: Process Visualization.* 8/41 (P = 2, A = 6) participants mentioned challenges due to shortcomings in the visualization of the process as a DFG. In line with the limitations outlined by van der Aalst [41], interviewees remarked that it is hard to derive insights based on the DFG since paths might be missing or indicate behavior that does not exist in the event log (e.g., *“I don’t trust the maps” [...] “because of this slider, we see paths, which already means you don’t see variants.” [...] “that’s not something that really happens”* or *“you cannot tell from the map how the process behaves because there are some paths missing”*).

All the respondents who validated this challenge (R = 11/24; P = 5, A = 6) understood it and 81.82% experienced it. The challenge occurs in the early phases of the analysis when presenting discovered DFGs to stakeholders who tend to misinterpret it due to the *“lack of properly visualized decision points”* or the complexity of cluttered “spaghetti-like” DFGs.

*C16: Analysis Experience.* 7/41 (P = 3, A = 4) participants reported that insufficient experience as a process mining analyst can complicate the analysis and put the initiative at risk. Interviewees said that if one *“doesn’t have much practice, the analysis is challenging in general.”* One participant elaborated that *“experience matters a lot. Experience means I have seen things before. Experience means I know that this approach will work. Experience means that I can start directly in the right direction and don’t try something that will eventually fail. [...] experience is a huge factor.”*

In the survey, 100% of the respondents (R = 6/24; P = 4, A = 2) understood and 33.33% experienced the challenge. The remaining 66.67% could relate to it. One respondent experienced this challenge when working on a project conducted by data engineers lacking experience in process mining analysis—a setting that made it harder to achieve the expected outcomes.

*C17: Analysis Focus.* The last challenge identified for this phase concerns the ability to keep the focus on the question/goal during an analysis. 6/41 participants (P = 6), all practitioners, told us that *“diving deep into one specific*” [aspect] *“but actually losing the big picture”* is a risk. It can easily happen that analysts *“lose themselves too quickly into details.”*

8/24 (P = 5, A = 3) respondents assessed this challenge, of which 75% understood it and 50% already experienced it. Interestingly, it was highlighted that losing the analysis focus *“is not necessarily something bad. If the focus is not lost completely and small deviations happen, I would rate this as helpful. However, bigger deviations from the original plan should be collected and funneled for future work.”*

#### Stakeholder evaluation

After conducting the analysis, analysts present their results to the stakeholders [12]. The two challenges identified for this phase, shown in Table  7, were derived from the statements of 11/41 (P = 7, A = 4) interviewees and validated by 10/24 (P = 9, A = 1) respondents.

**Table 7:** Overview of challenges identified for the *Stakeholder Evaluation* phase

	ID	Challenge	Description and Quote	Int. No.	Evaluation		
					No.	Und.	Exp.
	C18	Conclusions and Question Answering	Answering the question and completing the analysis is challenging, as it is not trivial to draw correct conclusions based on the results of process discovery	8	7	6 86%	4 57%
			*“After analyzing for a while, I don’t really know where to find the answers. I don’t know how to answer to the given question. This is actually a common problem.”*				
	C19	Recommendations and Next Steps	Giving recommendations and proposing next steps based on the results of a process mining analysis is challenging, as formulating concrete recommendations or deriving concrete next steps to foster process improvement is not straightforward	4	8	8 100%	5 63%
			*“The issue is really: What are you going to do with the results? What kind of recommendations or proposals to change the process can you really give based on it?”*				

*C18: Conclusion and Question Answering.* The analysis of an event log is supposed to lead to insights about the process based on which it should be possible to answer the initially raised questions [12, 21]. However, 8/41 (P = 4, A = 4) interviewees reported that it is difficult to *“come to hard conclusions or find what we should really change now.”* Additionally, it is challenging to identify correct conclusions: *“You can jump to conclusions which might not be relevant, and there are a lot of pitfalls where you can jump into some incorrect conclusion.”*

In the survey, the challenge was assessed by 7/24 (P = 6, A = 1) respondents, mainly practitioners. 85.71% of them indicated understanding the challenge and 57.14% having experienced it. Next to comments confirming that this challenge typically occurs during the late phases of a project, one respondent stated that drawing concrete conclusions is unlikely the result of a single analysis iteration: *“If you just try to answer a question, you probably won’t come up with an optimization potential directly. Properly completing the analysis requires a lot of creative thinking and deep understanding to simplify problems and reduce complexity.”*

*C19: Recommendations and Next Steps.* The second challenge of this phase concerns deriving concrete next steps for process improvement initiatives based on the analysis. We separated this aspect from C18 as it does not concern the act of “drawing conclusions” but rather focuses on coming up with *“recommendations or proposals to change the process.”* The challenge was raised by 4/41 (P = 3, A = 1) interviewees, who remarked that *“it’s challenging to answer with recommendations of what to do afterward.”* Indeed, process mining shows *“where issues are, but it’s not helping to solve them.”*

8/24 (P = 7, A = 1) respondents indicated understanding the challenge and 62.5% reported having experienced it in their projects. However, none of the respondents provided additional feedback on this challenge.

#### Challenges ranging across all phases

The remaining four challenges, presented in Table 8, are not tight to a specific project mining phase, either because the interviewees were not explicit about when they occurred or because different interviewees provided different insights, suggesting that they can occur at multiple moments in a project. Therefore, we named them as “overarching.” These challenges were initially raised by 34/41 (P = 19, A = 15) interviewees and subsequently validated by 21/24 (P = 12, A = 9) survey respondents.

**Table 8:** Overview of challenges ranging across all process mining project phases

	ID	Challenge	Description and Quote	Int. No.	Evaluation		
					No.	Und.	Exp.
	C20	Process Domain Understanding	It is challenging to understand the process domain. Analysts might not be familiar with the domain or domain-specific terminology used in the event and attribute descriptions	22	17	16 94%	14 82%
			*“I found it challenging, not understanding the background, the rules, the entire process - how it should work. I was confused by the domain-specific terms and the activity names used.”*				
	C21	Collaboration with Stakeholders	Collaboration with stakeholders in process mining projects is challenging, as there can be mismatching expectations, different process perspectives, different backgrounds of the stakeholders and the analysts, or stakeholders might be reluctant to participate in the success of a process mining project	15	9	6 67%	3 33%
			*“The challenge in the project was that I was hitting a wall with the Stakeholders. They didn’t want that somebody external to their business puts his eyes on it.”*				
	C22	Business Process Complexity	(Real-world) Business processes are often complex and therefore hard to analyze with process mining, due to their inherent complexity and dependencies among them	10	17	15 88%	11 65%
			*“My experience is that it is really easy to get tricked into believing that you found something interesting, but it is just due to the very complex process behavior.”*				
	C23	Enablement and Training	Enablement and training in process mining is challenging because training opportunities are not available, optimal courses are hard to find, and designing training courses is difficult since knowledge from different areas needs to be integrated	9	12	10 83%	8 67%
			*“Training is probably the most challenging aspect: To enable stakeholders on the methodology, but also on the operational side.”*				

*C20: Process Domain Understanding.* With 22/41 (P = 13, A = 9) participants, the challenge of understanding the process domain is supported by more than half of the interviewees. They highlighted that *“without domain knowledge, you won’t achieve much”* and stressed that understanding the domain-specific language (i.e., event and attribute descriptions) is difficult. Also, increased domain knowledge supports the analysis process (*“If you have domain knowledge, you know which path to check first”*) and helps in the evaluation with stakeholders (*“Often it is quite difficult to evaluate these results with the domain experts and to get the domain knowledge to explain what is happening in the data”*).

94.12% of the respondents (R = 17/24, P = 9, A = 8) indicated understanding the challenge and 82.35% having experienced it. While the described situations are supporting the assumption that the challenge spans across all project phases, it seems that it is often observed early in the project, already during the *Define Research Question* or *Data Collection* phases. Two respondents pointed out that they consider acquiring domain knowledge as the core (and *fun*) part of a project: *“The challenge always happens at the beginning of a project. However, this is also the fun part, to solve the puzzles.”*

*C21: Collaboration with Stakeholders.* When running a project, process analysts need to interact with different stakeholders to derive relevant questions, collect data, or evaluate insights [21, 46]. Although the availability of stakeholders and their collaboration is a success factor for projects [27], 15/41 (P = 9, A = 6) participants reported issues with such collaborations. They stated that *“communicating effectively what process mining can and should do to people from businesses is maybe the biggest challenge.”* The challenge originates from the different backgrounds of the stakeholders as well as hurdles caused by the reluctance of stakeholders to collaborate (*“They don’t want that somebody external to their business puts his eyes on it.”*).

Out of 9/24 (P = 5, A = 4) respondents, 66.67% confirmed understanding the challenge and 33.33% having experienced it. Another third (33.33%) indicated relating to the challenge without having experienced it. Respondents reported that divergent expectations among stakeholder groups make collaborations challenging, either because *“customers do not really know what they want or can expect from a process mining project”* or because *“they do not understand the purpose of having process mining. It certainly makes the project more complicated, especially when the business isn’t willing to collaborate or keeps pushing back on suggested improvement areas.”*

*C22: Business Process Complexity.* Challenges related to the interplay between departments, manifold IT landscapes, and the resulting complexity of the executed processes, were stated by 10/41 (P = 5, A = 5) participants. They reported a mismatch between the expectations regarding the analysis and its outcomes, as *“real processes, with several process objects, are more complex than the standard process coming from the vendors.”* The challenge was mainly mentioned in the context of the first (*Define Research Question*) and fourth (*Mining and Analysis*) project phases.

88.24% of the survey respondents (R = 17/24; P = 11, A = 6) indicated understanding this challenge and two-thirds (64.71%) having experienced it. Respondents also pointed out that process mining allows them to master this complexity and to get insights about it: *“I do see this as a challenge, but that is why the project is there in the first place. If this would be easy there would be little need for process mining.”* One of the respondents additionally reported that *“this is a tricky challenge since it might not be visible until very late in a project when the analysis is almost over and someone takes the full helicopter view.”*

*C23: Enablement and Training.* This challenge covers two main aspects that were raised by 9/41 (P = 6, A = 3) interviewees. On the one hand, participants addressed the problem of missing access to training and support in identifying (proper) training courses. A participant stressed that training in process mining should be better promoted. On the other hand, difficulties in the design of training for colleagues or students were raised. It was pointed out that it is especially challenging to train novices *“in many different fields to have some kind of basic understanding of how process models look like and, basic algorithms and not to misinterpret the process models that you get at the beginning, like DFGs.”*

Out of 12/24 (P = 6, A = 6) respondents, 83.33% confirmed understanding the challenge and 66.67% indicated having experienced it. Interestingly, while some claimed that *“there is lack of training materials for advanced topics in process mining”* (e.g., declarative process mining, causal process mining), others believe that training opportunities are *“widely available.”* Our respondents also pointed out the need for greater awareness of the change management aspect in the enablement of stakeholders (*“It is a long-term challenge, mindsets cannot be switched overnight. But having the relevant people to drive change management helps accelerate the process.”*).

In summary, our interviews revealed 23 challenges that were confirmed and enriched by the results of the questionnaire survey. Based on the statements of interviewees and respondents, we were able to capture the different nuances of each challenge and could underpin them with sample quotes. We found that 19 of the challenges were related to specific phases of process mining projects [12], while four remained overarching. We did not discover any significant correlation between the challenges and the expertise or experience levels of the people who reported or confirmed them. However, when differentiating the different role profiles of the interviewees and respondents, we noticed that challenges *C3: Process Mining Suitability*, *C6:Data Access*, *C7: Source System and Data Structure Knowledge*, *C17: Analysis Focus*, and *C19:Recommendations and Next Steps* are mainly perceived by practitioners. During the evaluation, C19 was also mainly confirmed by practitioners and seems indeed more relevant for practice, while the remaining four challenges received confirmations also from academics. Further patterns did not emerge.

In Sect. 5, we discuss how the discovered challenges relate to existing work and how the perception of analysts compares to challenges on the organizational level.

### Perceived relevancy of process mining challenges

In this section, we present the results for RQ.2 and report on the relevancy assessment of the challenges and the extent to which they can be solved in practice. Thus, we provide a possibility to prioritize and differentiate the challenges along different dimensions.

The *Project Blocker* and *Criticality* dimensions reveal the extent to which a challenge puts a process mining initiative at risk, while the *Frequency* dimension provides information on whether the challenge is frequently occurring or exceptional. A critical challenge that is perceived as a project blocker but only occurs in rare cases is potentially less relevant than frequently occurring challenges. In addition, the analysis of whether a challenge can be *solved* in practice hints toward the existence of mitigation strategies.

**Fig. 5:**
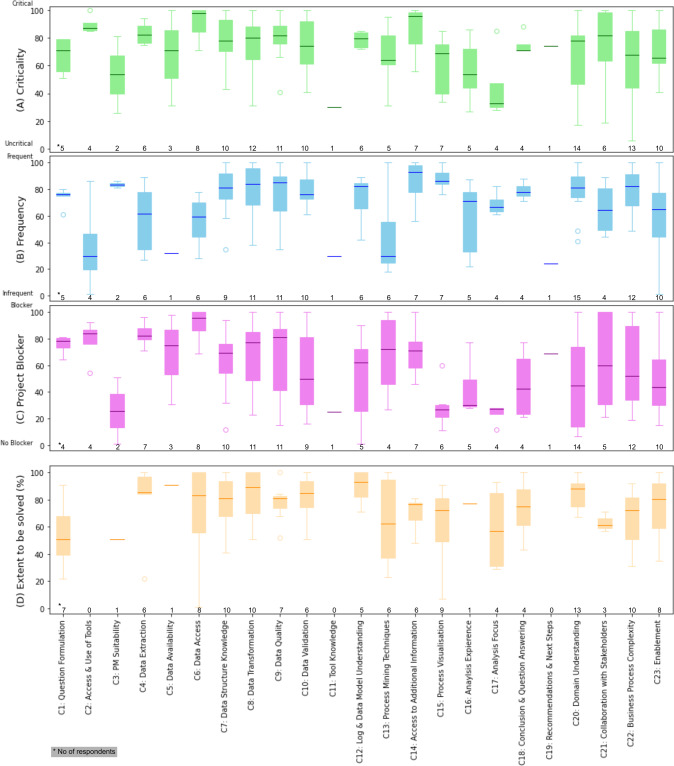
Distribution over relevancy ratings per challenge for **A** Uncritical versus Critical, **B** Infrequent versus Frequent, **C** No Blocker versus Project Blocker and **D** Extent to which the challenge could be solved in practice. The number of responses is indicated for each dimension and challenge

**Fig. 6:**
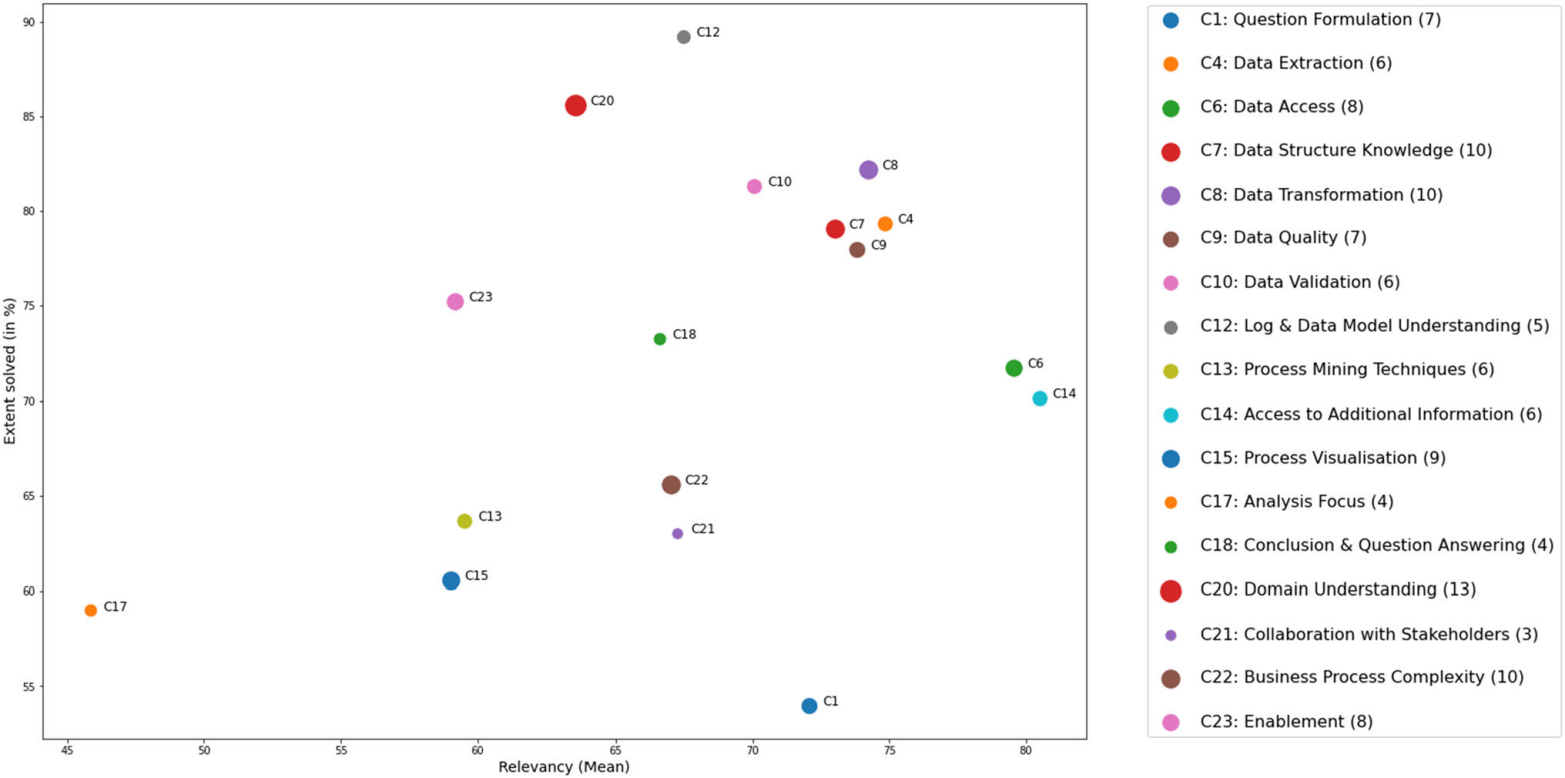
Scatter plot showing the average relevancy in relation to the extent to which respondents indicated they could solve the challenge. The size of the scatters indicates the number of respondents who provided an assessment (the absolute number is reported in the legend). Challenges that received at most one reply are excluded

An overview of the ratings of the three relevancy dimensions and the solved-ratings per challenge is presented in Fig. 5. With an average rating of 80.48 over the first three dimensions (cf. Fig. 5A–C), challenge *C14: Access to Additional Information* is perceived as the most relevant one. It is followed by *C6: Data Access* ($mean\,=\,79.56$) and *C4: Data Extraction* ($mean=74.82$). Two of the 23 challenges, namely *C11: Tool Knowledge* ($mean=28.33$) and *C17: Analysis Focus* ($mean=45.83$), received average relevancy ratings of less than 50 (out of 100), indicating that they are less relevant for practice. Particularly for C11, respondents provided little feedback during the survey and only indicated that one has to learn how to use the tools. No one rated the extent to which the challenge could be solved.

As presented in Fig. 5D, challenges *C5: Data Availability*, *C12: Event log and Data Model Understanding*, and *C20: Domain Understanding* are the three challenges that can be solved to the largest extent. With ratings of 91%, 89.2%, and 85.62%, the respondents indicated that they could apply mitigation strategies that helped them overcome the challenges in their projects to a large extent. We will lay out further details about these strategies in Sect. 4.3. We did not receive feedback regarding all challenges for this dimension but challenges *C3: Process Mining Suitability* ($mean = 51\%$), *C1: Question Formulation* ($mean = 54\%$), and *C17: Analysis Focus* ($mean = 59\%$) are the three that turned out to remain largely unresolved in practice.

In Fig. 6, we show the dependencies between the relevancy (average of dimensions (A) Criticality, (B) Frequency, and (C) Project Blocker) of a challenge and the extent to which it can be solved in practice. As one can see, both ratings vary across challenges, and with a correlation of 0.34 a common trend is not apparent. Interestingly, challenges connected to the understanding of the analyst, such as challenges *C12: Event Log and Data Model Understanding*, *C20: Process Domain Understanding*, and some of the challenges occurring during the *Data Collection* (i.e., C4, C7) and *Data Preprocessing* (i.e., C8–C10) phases could be solved to a larger extent by the respondents. For example, *C3: Data Extraction*, which is rated as one of the most relevant challenges, could at the same time be solved by more than 80%. On the other hand, challenges *C14: Access to Additional Information*, *C21: Data Access*, and *C1: Question Formulation* are perceived as relevant and remain rather unresolved. In general, challenges that remain for the most part unresolved seem to be challenges that are either linked to external factors outside of the control of analysts, such as the three challenges just mentioned, or are rather technical challenges, hinting at missing tool functions or limited methodological support (e.g., *C13: Process Mining Techniques*, *C15: Process Visualization*, *C22: Business Process Complexity*).

### Mitigation strategies

To address RQ.3, we analyzed the mitigation strategies that the survey respondents applied in their projects when they encountered challenges. Based on the 115 replies collected across all challenges, five key areas of mitigation strategies emerged. A summary of them is presented in Table 9. As we asked the respondents how they overcame a specific challenge, the mitigation strategies are linked to the challenges they were reported for.

**Table 9:** Summary of mitigation strategies

Main goals	Linked challenges	Concrete aspects
**Mitigation Strategy 1: Involvement of Stakeholders**		
(1) Increase understanding	C4, C7, C8, C12, C16, C20, C22	e.g., Executives, Business, Process Owner, IT, Domain Experts
(2) Alignment of expectations	C1, C4, C6, C10, C14, C21, C22	
(3) Identification of (key) requirements	C1, C7, C8, C9, C10, C16	
**Mitigation Strategy 2: Creation or Usage of Artifacts**		
(1) Gather information	C1, C7, C8, C10, C12, C14, C20, C23	e.g., Code Snippets, Analysis Templates (Dashboards), (online) documentations, Sample Cases, Training Material
(2) Persist or share information	C4, C6, C17, C22	
**Mitigation Strategy 3: Following an Iterative and Pragmatic Approach**		
(1) Minimize involvement of stakeholders	C14	e.g., Iterations between validation with stakeholders and analysis, Trade-off between full understanding and pragmatic approach
(2) Increase understanding	C1, C7, C8, C10, C12, C18, C22	
(3) Trade-off between “perfection” and pragmatism	C7, C10, C22	
**Mitigation Strategy 4: Usage of Specific Techniques**		
(1) Replace non-existing functionality with another tool	C4, C8, C9, C13, C15	e.g., Simulation, Delta Loads, Replication Databases, Anonymization/Pseudonymization, Coding (e.g., Python), DECLARE, Petri nets, Filtering, Clustering, Aggregation
(2) Get access to (more) data	C5, C6	
(3) Use process mining functionalities to reduce complexity	C9, C15, C22	
**Mitigation Strategy 5: Creation of Conceptual Models**		
(1) Increase understanding	C7, C8, C12, C20	e.g., Domain Model, Data Model, Process Model
(2) Communicate understanding	C12	

*Involvement of Stakeholders.* For 15 of the 23 challenges, respondents pointed to the involvement of stakeholders for mitigating several obstacles. In these cases, stakeholders can be different parties, individuals, or groups who, for different reasons, should either be involved after a challenge has arisen, involved in the decision-making early in the project, or informed of particular procedures from the outset to avoid challenges later on. Particularly in the case of problems due to missing information or when there is a need for the analyst to increase their understanding about something, the involvement of stakeholders can be helpful. However, as these aspects also explicitly emerged in *C14: Access to Additional Information* and *C21: Collaboration with Stakeholders*, this strategy might not be applicable to all projects. As part of the mitigation strategy, respondents indicated that it is advisable to align expectations from the outset of the project and clarify information and collaboration needs from the beginning as this might help, next to others, to mitigate C14 and C21. As a last instance, several respondents had to involve the project sponsor or executives to increase the involvement of business stakeholders (cf. C21) or to receive access to the data (cf. C6).

*Creation or Usage of Artifacts.* Many respondents mentioned the use of existing artifacts or the creation of new ones to mitigate 12 challenges. Artifacts in process mining projects can be manifold and include templates, documentation, existing guidelines, or online materials.

When using artifacts to overcome challenges, they often serve as a starting point and then need to be refined further. For example, for challenge *C1: Question Formulation*, respondents suggested starting with generic insights based on a discovery template (dashboard) created by experts to subsequently derive more meaningful questions. Thus, next to a joint working mode with stakeholders (Mitigation Strategy 1) the usage of templates can support analysts in deriving questions. Similarly, for challenges *C8: Data Transformation*, *C10: Data Validation*, *C14: Access to additional Information*, and *C23: Enablement and Training*, respondents advised starting from templates, available documentation, or standards and refining them based on their specific needs.

For other challenges, it turned out to be helpful to create custom artifacts. For example, to avoid losing focus during the analysis (cf. C17), the creation of a backlog with an outline of pending tasks can assist analysts in keeping an overview. This enables new insights to be set aside for later consideration, rather than pursuing them immediately and shifting the focus.

However, although we discovered that it can be beneficial to use artifacts for several process mining tasks to ease challenges, we also acknowledge that there are limitations. Artifacts might not be available (cf. C14) or require a high level of expertise (cf. C16) to be created.

*Following an Iterative and Pragmatic Approach.* For nine of the challenges, respondents highlighted the usefulness of adopting a certain approach or method to mitigate challenges. A main theme that emerged revolves around iterative work practices. Many challenges can benefit from iterations between the identification of results, findings, or questions and the subsequent consultation and discussion with relevant stakeholders, such as business users. This indicates that the working mode applied by the analysts can have a positive impact on challenges.

Additionally, respondents pointed out that aiming for “perfection,” for example, by achieving high data accuracy (cf. C9 and C10) or full domain (cf. C20) and source system understanding (cf. C7), might not be required in all cases. They suggested considering trade-offs between deep understanding and a time- and resource-efficient approach. Note that a more pragmatic approach to tackle process mining tasks does not provide a solution to connected challenges, but rather decreases their criticality in practice.

*Usage of Specific Techniques.* For eight challenges (mostly but not exclusively those related to the *Data Collection* and the *Data Preprocessing* phases), mitigation strategies involving the use of specific techniques and technologies are suggested. The proposed techniques are quite diverse and differ across challenges. For example, for *C15: Process Visualization*, respondents advised alternatives to employing DFGs. These include utilizing DECLARE models [10] or Petri nets [40], to, for example, better capture concurrency and avoid the representation of non-existing transitions from the event log. Another recommendation is to simplify the DFG by aggregating activities or applying filters during the analysis to increase its understandability. Although this might help practitioners in certain settings, creating different process models requires good expertise and access to tools (cf. C2, C16) and does not solve general shortcomings of the DFG [41]. Other strategies suggested to conquer challenges during the *Data Collection* and the *Data Preprocessing* phases, such as incremental data loads, data simulation, or anonymization, are not easy to implement and require engineering skills that novices might not have.

*Creation of Conceptual Models.* The last mitigation strategy that emerged is the creation of conceptual models. We consider conceptual models to be any representation created by the process mining analyst to structure, clarify, or illustrate an aspect of the process. We observed that the use of conceptual models during process mining projects can help to increase the understanding of the analyst and summarize the available information. In particular, the use and creation of conceptual models, whether through diagrams or notes, seems to help mitigate four of the challenges (i.e., C7, C8, C12, C20). For *C8: Data Transformation*, it was additionally pointed out that *“creating the event log is not foremost a technical challenge, which is well-understood. It is a model-building challenge (what objects to consider as cases, what event is relevant, how can I detect the event, what is the proper label), which is always use case- and domain-specific.”* Hence, analysts often underestimate the significance of possessing a clear conceptual model.

In total, we identified mitigation strategies revolving around five themes. All can provide good starting points for analysts and should be considered in projects. However, none of the challenges could be fully solved by the application of any of the strategies and many strategies are very specific to the context of the project and the possibilities and skills of the analyst.

## Related work

In this section, we first present related research on challenges, gradually narrowing our focus to studies that present process mining challenges (cf. Sect. 5.1). Then, we map the challenges discovered in this work, i.e., challenges of individual process mining analysts, against challenges discovered in closely related work (cf. Sect. 5.2). This mapping highlights commonalities and significant differences between our work and the related literature and shows where the individual perspective adds to the existing body of knowledge.

### Overview of related work

*Challenges in Data Analysis.* Before looking at the body of the literature in the process mining area, we report on a few papers that address challenges in the broader field of data analysis. Since several tasks within process mining projects are comparable to general data analysis operations [46], we consider the comparison with works in this area to be relevant. However, important differences such as the available tools, the novelty of process mining as a discipline for many organizations, and the specific type of required data (event log format), make a direct comparison of challenges intricate.

Kandel et al. [16] report the results of an interview study with 35 analysts, providing insights into the process of industrial data analysis as well as existing challenges and adoption barriers. Similar to our work, the authors identified challenges related to the collection and preparation of data that emerge in the day-to-day practices of data analysts. In particular, they point to difficulties comparable to challenges C4 and C6–C9. Additionally, they also uncover challenges comparable to *C17: Analysis Focus*, as analysts tend to lose track of the operations they performed and their rationale.

Not considering the individual analyst but focusing on the organizational level, Vidgen et al. [48] conducted a Delphi study and interviews with business analytics experts and managers to identify challenges for organizations in creating value from their data. The authors present 31 challenges and 21 recommendations for organizations to enhance business value creation using data analytics techniques. While often beyond the control of individual analysts, aspects such as having a clear corporate analytics strategy, informed technology selection, and clarity regarding regulations and legislation of data are likely to have a positive impact on individuals operating within organizations. Challenges C2, C14, C19, C20, C21, and C23, as well as challenges occurring during the *Data Collection* and the *Data Preprocessing* phases (i.e., C4–C7, and C9), relate to aspects discussed in [48].

*Challenges in Process Mining.* In the area of process mining, several papers have reported on challenges in different contexts.

A first bucket of the literature includes community-led research focusing mainly on challenges for process mining research. Prominent examples are the *Process Mining Manifesto* [43] or the recently published *Process Mining in Healthcare Manifesto* [30]. Also in [4], among the nine problems observed in the broader area of business process management, several aspects can be attributed to challenges in process mining. Examples are the problems of dealing with process activities having a fixed granularity or augmenting process mining with common sense and domain knowledge, which relate to C8 and C20. While these papers touch upon challenges in process mining, they refer to general challenges that remain open in the research field and do not report on challenges occurring in the daily practices of process mining analysts.

Another group of works more closely related to ours are case studies, in which challenges are derived from the practical application of process mining to specific settings. For example, Dakic et al. [8] analyze a manufacturing event log to compare two process mining tools (i.e., ProM [45] and Fluxicon Disco [14]) and validate some known problems in the field. Similarly, Sterz et al. [38] look into the application of process mining in manufacturing companies and complement their findings with insights into the benefits and drawbacks of process mining gained through focus group interviews. However, only a few case studies explicitly report on challenges and provide accurate descriptions of them.

Among them, Eggert and Dyong [11] apply process mining in an IT organization and identify 13 challenges and seven guidelines they retrieved from the application of process mining and additional interviews with involved stakeholders. Smit and Mens [37] focus on the application of process mining for different use cases in a rail organization and also support their observations with interviews. They report challenges in three different areas. Syed et al. [39] analyze the application of process mining in an organization that just started to adopt process mining. They identify seven challenges observed during the adoption by end-users.

In addition to case studies, empirical works that analyze the adoption of process mining and explicitly report connected challenges exist. Thereof, the closest to our work is the paper from Martin et al. [29], who conducted a *Delphi study* to identify opportunities and challenges for organizations in applying and adopting process mining. Also, Kipping et al. [19] and Grisold et al. [13] report on challenges they observed for the adoption of process mining in organizations.

The latter six papers [11, 13, 19, 29, 37, 39] provide an explicit reporting of challenges emerging from applying process mining in practice and are therefore comparable to our work. They are summarized in Table 10. None of them focuses on the needs and the perception of individual analysts, but all six are concerned with the organization, i.e., the adoption of process mining in organizations or its specific application in certain domains.

**Table 10:** List of papers explicitly reporting on challenges in process mining

References	Authors	Title	Method	Focus	No. challenges
[11]	Eggert & Dyong	Applying Process Mining in Small and Medium Sized IT Enterprises—Challenges and Guidelines	Case Study	Use in IT organizations	13
[13]	Grisold et al	Adoption, use and management of process mining in practice	Focus Groups	Adoption and use in organizations	10
[19]	Kipping et al	How to Leverage Process Mining in Organizations—Toward Process Mining Capabilities	Interviews	Adoption and use in organizations	4
[29]	Martin et al	Opportunities and challenges for process mining in organizations: Results of a Delphi study	Delphi Study	Adoption and use in organizations	32
[37]	Smit & Mens	Process Mining in The Rail Industry: A Qualitative Analysis of Success Factors and Remaining Challenges	Case Study	Application in rail industry	3
[39]	Syed et al	Process Mining Adoption	Case Study	Adoption in organizations	7

### Mapping of challenges

To show where the perspective of our work (i.e., the focus on the perception of individual analysts) adds to the existing body of knowledge, we mapped our challenges to the findings of the papers listed in Table 10. To this end, we derived a list of the challenges and their descriptions from each paper. As the challenges from other studies were collected in different contexts, with the help of different methodologies, and the authors aimed to answer different research questions, such a mapping is not trivial. Therefore, we differentiate between a full  or partial mapping and further discriminate partial mappings that are due to the different level of granularity of the challenges  from those due to a different perspective or focus .

The detailed mapping is presented in Table 11. The overview reveals that we identified nine novel challenges that are not mentioned in related work. They cover aspects connected to the understanding of the analyst (i.e., C7, C12), to formulating and answering questions (i.e., C1, C18, C19), to planning and conducting the analysis (i.e., C3, C14, C17), and to validating the data (i.e., C10). While the related work addresses seven additional challenges, it does not encompass all the aspects we have discovered (partial mappings). This concerns the two most supported challenges of the *Data Collection* and the *Data Preprocessing* phases (i.e., C4, C8), challenges connected to the expertise of the analyst and respective enablement (i.e., C16, C23), and tool-related challenges such as supported techniques and shortcomings of process visualizations (i.e., C11, C13, C15). For the remaining seven challenges (i.e., C2, C5, C6, C9, C20, C21, C22), we identified at least one full match with related work. Interestingly, no challenge is mentioned in all identified papers, and among the six related works we analyzed, our challenges intersect with a maximum of three papers. The highest number of overlapping challenges can be identified with the work of Martin et al. [29], for which seven partial matches and four full matches emerged.

The mapping shows that especially the challenges identified for the first (*Define Research Question*), fourth (*Mining and Analysis*), and fifth (*Stakeholder Evaluation*) project phases add to the existing body of knowledge. For the first phase, only *C2: Access and Use of Tools* is already considered while the other two add new aspects. For the fourth phase, we could partially map four of the seven challenges to related work. Martin et al. [29] also uncovered the problem of insufficient analytical skills (partially matches C16), the problem of incomprehensible outcomes (partially matches C15) and the lack of advanced features (partially matches C13), and Syed et al. [39] covered parts of C11 in their broader category of *“User-Related Challenges.”* However, none of these challenges fully encompass all the aspects we subsume under our challenges. Additionally, the two challenges we identified for the *Stakeholder Evaluation* phase have not yet been discovered by any of the related work. Instead, challenges identified for the *Data Collection* and the *Data Preprocessing* phases as well as the overarching challenges are covered to a larger extent, indicating that they are less specific to the individual level.

By focusing on the perception of analysts, we have not only uncovered several new challenges but also introduced novel perspectives to existing ones. Our work enhances the existing literature by adding more details and context to the challenges and quoting the voices of analysts (cf. Sect. 4.1). Additionally, we supplemented the challenges with a relevancy assessment (cf. Sect. 4.2) and corresponding mitigation strategies (cf. Sect. 4.3).

## Discussion

In this paper, we analyzed the challenges that are perceived by *individual* process mining analysts. After having identified and validated 23 challenges, we assessed their relevancy and the extent to which they have been solved by our interviewees and survey participants. Additionally, we reported on mitigation strategies that analysts follow to deal with them in practice. We discuss the main implications of our findings for process mining research and practice in Sect. 6.1 and the limitations of our work in Sect. 6.2.

**Table 11:**
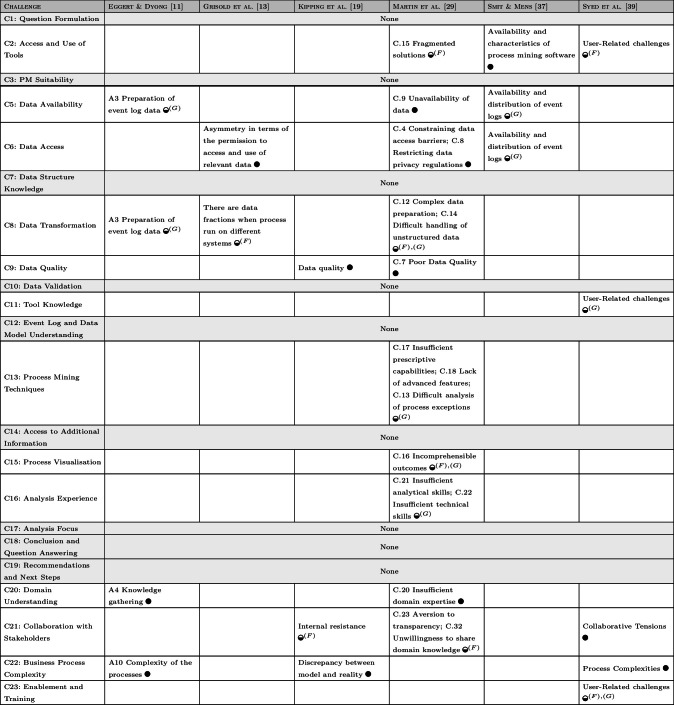
Mapping the challenges discovered in this paper to the articles described in Table 10

### Implications for process mining research and practice

#### Challenges as a motivation for method and tool development.

The extensive catalog of challenges provided in this work serves as a basis for deriving requirements for the development of methodological and tool-based solutions to support process analysts in their work. The nuanced description of the challenges and the context in which they occur provide insights into the specific tasks and application scenarios where analysts still perceive a lack of adequate support from existing tools. For example, for *C1: Question Formulation* participants reported that it is challenging to conduct analyses without questions as well as to formulate “good” questions at the beginning of a project. However, for both problems, tool support and methodological guidance are still limited. Based on the rich insights from the interview study, we have suggested a concrete extension of existing methodologies to provide recommendations that analysts can follow to formulate questions *during* an analysis [53]. Similarly, our work provides empirical evidence of actual user needs and can inspire solutions for other challenges, such as *C17: Analysis Focus* or *C12: Event Log and Data Model Understanding*, as demonstrated by Zerbato et al. [52] or for *C18: Conclusion and Question Answering*, as addressed in [49]. Also in the case of challenges concerning techniques already implemented in process mining tools, such as *C13: Process Mining Techniques* and *C15: Process Visualization*, the perceptions of individual analysts serve as a valuable form of explicit feedback, exposing areas where tool design should be improved.

#### A call for solutions on different levels of process mining research.

As pointed out in Sect. 5, our findings complement existing literature focusing on process mining challenges from a technical [30, 43] and an organizational perspective [13, 29]. The direct comparison with challenges elicited in closely related work (cf. Table 11) shows that many of the challenges we identified—despite being perceived at the individual level—also play a role on other levels of the process mining research framework [50] (cf. Sect. 2). For example, *C20: Domain Understanding* is not only a challenge for individual analysts, but is also recognized by managers in an organizational context [29] and is necessary for the interpretation of process mining results in IT enterprises [11]. Since challenges manifest themselves on different levels, we argue that not all of them can be solved on the individual level alone but that solutions might be found on the other levels of process mining.

**Technical level:** Looking at the challenges, it is noticeable that several of them relate to the *technical level* and that solutions could start from implementing new tool functionalities. For example, challenges C13 and C15 concern missing or inadequate techniques. Also, difficulties in deriving conclusions (C18) hint at the need for better root cause analysis techniques. Indeed, although research around automated root cause analysis in process mining exists (e.g., [22, 32, 47]), many of our interviewees still reported struggling with deriving conclusions. Besides, especially *Mitigation Strategy 4: Usage of Specific Techniques* is closely linked to the technical level. Analysts tend to leverage additional tools or standard filtering functions to address data-related challenges such as C4, C5, C6, C8, and C9, but also challenges such as C15 which does not refer exclusively to the technical level, but includes user understanding.

**Group level:** *C21: Collaboration with Stakeholders* is a frequently perceived challenge that relates to interactions in teams (cf. the *group level* in [50]). While being identified as challenging, the collaboration and involvement of stakeholders emerged as a commonly applied mitigation strategy for numerous other challenges (cf. Mitigation Strategy 1). In this case, solutions to challenges related to stakeholder collaboration could start at the group level and explore how to assemble teams and how to best organize them to enable effective interactions in process mining projects.

**Organizational level:** At last, some of our challenges (i.e., C2, C4, C5, C6, C8, C9, C11, C20, C21, C22) relate to the *organizational level* and have already been identified in related literature on process mining adoption. This is not surprising, as limited adoption in the organization also affects the individual analyst, whose work can be hampered by inadequate governance structures. Although we found that analysts use specific techniques or artifacts to mitigate data-related challenges (C4, C5, C6, C8, C9), stronger organizational measures like the setup of company-wide standards and training could additionally serve as solutions on the *organizational level*. In this context, the recommendations put forth by Vidgen et al. [48] to facilitate the adoption and value creation of process analytics in general, along with the more specific recommendations for process mining proposed by Eggert and Dyong [11], could serve as a starting point for improving the application within organizations. Measures on the organizational level could also help to address challenges C1, C18, and C19 which relate to the ability of analysts to provide value-adding process insights to their organizations.

#### Mitigation strategies as an opportunity for practical solutions.

The mitigation strategies presented in this paper provide practical advice for specific tasks across different project phases. For process mining practice, mitigation strategies can serve as workarounds in settings where mature solutions are still lacking. This is especially true for novice analysts who have not experienced certain challenges and workarounds to circumvent difficulties that arise in their work practice. For process mining research, mitigation strategies offer a concrete starting point for best-practice solutions. For example, our findings in Sect. 4.3 show that experienced analysts apply mitigation strategies revolving around methodological approaches (Mitigation Strategy 3) and conceptual models (Mitigation Strategy 5). To the best of our knowledge, these aspects are not yet systematically integrated into existing methodologies. Although, for example, the well-known PM^2^ methodology [46] prescribes multiple analysis iterations, the creation and refinement of conceptual models or the involvement of stakeholders throughout the analysis are not defined in detail. This is one example where less-optimal solutions such as mitigation strategies could be leveraged into best-practice solutions to support individual analysts in reasoning and decision-making during process mining projects.

### Limitations

We followed an empirical approach in our work, retrieving results from interviews and subsequently evaluating them through a questionnaire survey. Despite our efforts to minimize limitations, certain aspects should be considered when interpreting the results. First, we only present challenges explicitly stated by the interviewees. Even though we conducted a large number of interviews, our findings might not be complete as people might not have reflected on all the challenges they experienced in their work practice. Second, the perception of a challenge may be subjective and participant-dependent. To obtain valid and reproducible results, we excluded all the challenges mentioned by less than four individuals. Third, the interviews were conducted right after an analysis task, potentially introducing a task-specific bias. The evaluation of the challenges by a second data collection confirms that the bias if it exists, must be low, as all the challenges have been confirmed and experienced in practice by at least one questionnaire respondent. Lastly, the evaluation of the 23 challenges resulted in a rather long questionnaire, requiring respondents to invest a significant amount of time to assess each challenge and to describe situations in which it occurs and the mitigation strategies followed to address them. Therefore, we reduced the time and effort with an adaptive questionnaire design and optional fields. This approach resulted in a varying number of replies per challenge, complicating the direct comparison of the relevancy of challenges. In our findings, we state the absolute number of replies and highlight the potentially limited expressiveness of the rankings reported by fewer respondents. As such, our findings could be complemented by a survey that only focuses on the relevancy of the challenges or a validation based on case studies.

## Conclusion

This work provides an overview of challenges perceived by process mining analysts. It goes into the details of the varying relevancy of these challenges and further outlines mitigation strategies that are applied by practitioners in cases where existing technologies and organizational governance structures are not yet advanced enough. Thus, our work provides relevant information for practitioners as these strategies can help to circumvent challenges. Furthermore, the insights of our work provide important directions for future research aimed at improving the field. Each of the identified challenges impedes the work of individual analysts, prevents them from working efficiently and effectively, and, in the worst cases, discourages them from undertaking further process mining projects in their organizations. While we focus on the individual perspective, we found that the challenges discovered also affect the organizational, group, and technical levels and require enhancement at these levels to be resolved.

## Footnotes

1 https://www.celonis.com/; accessed on 07 Aug 2023.

2 https://www.signavio.com/process-mining/; accessed on 07 Aug 2023.

3 https://powerautomate.microsoft.com/en-us/process-mining/; accessed on 07 Aug 2023.

4 https://dcrsolutions.net/dcr-process-mining/; accessed on 29 Aug 2023.

5 https://www.workfellow.ai/; accessed on 29 Aug 2023.

6 https://www.maxqda.com/; accessed on 09 Jan 2023.

7 https://www.linkedin.com/groups/1915049/; accessed on 26 Sept 2022.

8 https://www.gdpr.eu
